# Disease Burden and Costs Associated with Multiple Sclerosis in China: A Cross-sectional Analysis of Nationwide Survey Data

**DOI:** 10.1007/s12264-023-01135-5

**Published:** 2023-11-02

**Authors:** Yusheng Jia, Xuanqi Qiao, Jin Zhao, Hainan Li, Shanlian Hu, Min Hu

**Affiliations:** 1 Department of Health Economics, School of Public Health, Fudan University, Shanghai, 200032 China; 2 National Health Commission Key Laboratory of Health Technology Assessment, Fudan University, Shanghai, 200032 China

**Dear Editors,**

Multiple sclerosis (MS) is a chronic, autoimmune neurological disorder characterized by inflammatory demyelination of the central nervous system; it is associated with reduced mobility, cognitive impairment, and numerous comorbidities [1]. Global MS incidence (2.1 per 100,000 person-years) and prevalence (30.1 per 100,000) have increased in recent years, and its prevalence is notably higher in Western countries, ranging from ~30 to 200 cases per 100,000 [2]. In China, recent epidemiological research has shown that the crude prevalence was 2.44 per 100,000 in 2016 [3], and the age- and sex-adjusted incidence was 0.235 per 100,000 person-years [4]. Despite the relatively low prevalence compared with the global average, China has extensively addressed the well-being of over 30,000 MS patients, enrolling MS in China's First National List of Rare Diseases in 2018.

Evolving MS stages are correlated with deteriorating health-related quality of life (HRQoL), with disability, fatigue, and depression weighing heavily. The economic burden of MS has been studied in many high-income countries, and these studies highlight the cost escalation linked to MS progression and relapses [5, 6]. The chronic and progressive nature of MS not only contributes to increased healthcare expenditures but also results in an increasing need for long-term care, a caregiving burden, and productivity loss for households. The various dimensions of economic burden impose significant capability deprivation on patients' households. Informal caregivers are often required to deliver continuous care and support, resulting in increased burden and challenges for the families of MS patients [7].

Despite the substantial disease burden and costs among MS patients, there are very limited published data on either the HRQoL or comprehensive estimation of the economic burden of MS in China. There is currently no published literature on HRQoL among Chinese MS patients, and previous studies used health insurance claims data or hospital monitoring data to estimate outpatient or inpatient costs, which is only a partial cost estimation for MS patients [4, 8–10]. Furthermore, the connection between disability and costs in China's MS patients remains inadequately explored. One previous study identified factors contributing to high direct costs for MS patients, such as tertiary hospital admissions and length of stay [8], but it is unclear how the association between types of costs and disease severity may differ.

Exploring MS's economic burden in China carries broader implications for future research, such as population-based disease burden evaluations and cost-effectiveness analyses. This research is essential for shaping priorities in healthcare policy as China undergoes transformative healthcare system changes and escalating health expenditures. Moreover, with the rapidly changing health system and growing health expenditures in China, time-sensitive data on the cost of MS is urgently needed. In this study, we aimed to comprehensively estimate the disease burden and costs of MS and their association with the severity of disability. Our research bridges the gap in understanding the HRQoL of MS patients and the relationship between disease severity and various cost components. Moreover, our study pioneers a holistic evaluation of direct medical, direct non-medical, and indirect costs. These empirical insights are poised to inform critical public health decisions in China.

**HRQoL and Productivity Loss Due to MS**

A total of 477 MS patients were included in the study, with 14.7% involving family members as proxies (excluding the EQ-5D-5L scale). Questions based on the expanded disability status scale (EDSS) were applied to assess disability severity [11]. The results revealed 29.1% reporting a good or better health status, yet the quality of life declined significantly with disease progression. The average health utility calculated using the EQ-5D-5L scale was 0.657. However, for mild *versus* severe MS, utility values were 0.808 and 0.017, indicating health deterioration (see appendix).

The employment rate of the patients was 50.3%, with decreasing rates alongside disease progression. Unemployment was common among patients with severe MS. Concerning productivity loss due to MS, 47.6% of patients reported unemployment or early retirement due to MS, and the proportion of patients unemployed or taking early retirement due to MS increased as the EDSS score escalated (EDSS <4: 37.4%; EDSS 4–6: 63.9%; EDSS >6: 89.7%). Among the employed, the annual average of MS-related absence was 63.5 days.

**Costs Due to MS**

Figure 1 illustrates direct medical costs, direct non-medical costs, and indirect costs. Regarding direct medical costs, MS patients paid 61,976 CNY (8739 USD) per year for medical services. These costs decreased with escalating severity of disability (EDSS <4: 65,299 CNY; EDSS 4–6: 55,337 CNY; EDSS >6: 55,923 CNY). The mean out-of-pocket (OoP) expenditure (39,895 CNY; 5625 USD) constituted 64.4% of average direct medical costs, implying the potential financial hardship faced by the families of MS patients.

**Fig. 1:**
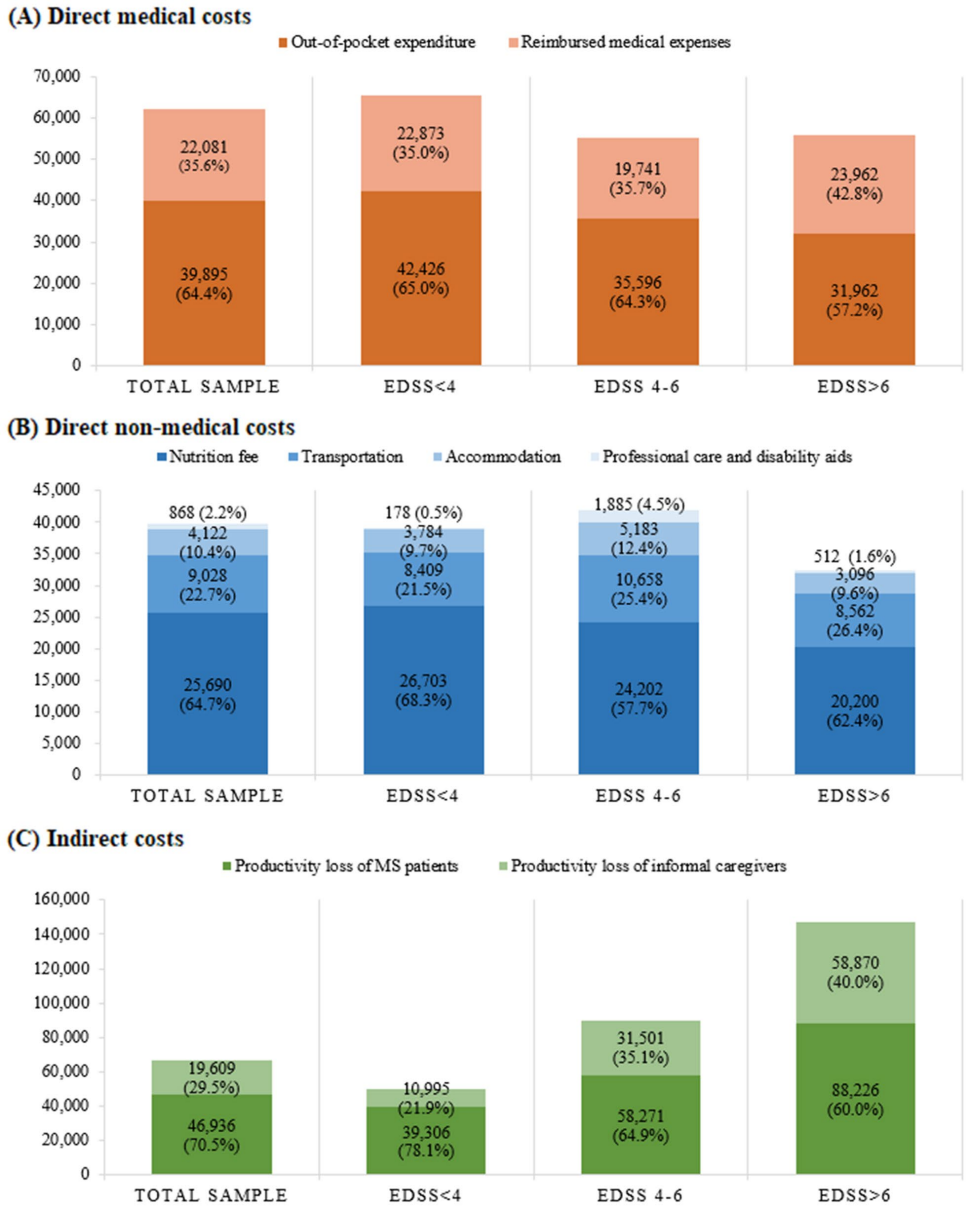
Costs of multiple sclerosis per year in CNY. **A** direct medical costs, **B** direct non-medical costs, and **C** indirect costs are shown according to the severity of disability, which is indicated by EDSS

The average direct non-medical costs were 39,708 CNY (5599 USD) annually, encompassing professional care, disability aids, transportation, nutrition, and accommodation. Nutrition fees accounted for the largest share (64.7%), followed by transportation costs (22.7%), while expenses related to professional care and disability aids were comparatively minor.

Indirect costs, driven by productivity losses among patients and informal caregivers, averaged 66,545 CNY (9383 USD) annually. Patient productivity losses dominated (70.5%), but with disease progression, caregiver productivity losses grew as a proportion of total indirect costs (EDSS <4: 21.9%; EDSS 4–6: 35.1%; EDSS >6: 40.0%).

On average, indirect costs accounted for 39.6% of the total, followed by direct medical costs (36.8%) and direct non-medical costs (23.6%). The mean annual total cost reached 168,228 CNY (23,720 USD). Notably, total annual costs were lower for MS patients with EDSS <4 (154,673 CNY; 21,809 USD) compared to those with EDSS 4–6 (187,038 CNY; 26,372 USD) or EDSS >6 (235,390 CNY; 33,190 USD).

Notes: Total direct medical costs were collected using a self-reported questionnaire, including outpatient consultation, inpatient visits, and treatment costs. The unemployment rate was calculated among MS patients of working age. The unemployment status was confirmed when the respondent had been suspended, unemployed, or retired early for more than one year. The average wage refers to GDP per capita in 2020 in China, which is 72,000 CNY per year (i.e., 197.26 CNY per day).

**Factors Associated with MS Costs**

Increasing disability severity was correlated with rising total costs linked to MS. The indirect costs had a significant association with disability severity (p <0.01), unlike the direct costs (Table 1). The share of indirect costs increased from 32.5% (EDSS <4) to 62.5% (EDSS >6) as disability worsened, whereas the direct costs remained relatively constant.

**Table 1:** Association between costs of MS and level of disability

Variables	(1)	(2)	(3)
	Direct medical costs	Direct non-medical costs	Indirect costs
*EDSS (ref: EDSS <4)*			
EDSS 4–6	0.02	0.22	1.87***
	(0.17)	(0.15)	(0.57)
EDSS >6	0.04	− 0.33	2.42***
	(0.25)	(0.24)	(0.63)
*Age group (ref: <30 years)*			
31–40	− 0.04	− 0.12	0.07
	(0.15)	(0.15)	(0.52)
41–50	− 0.06	− 0.35**	− 0.20
	(0.17)	(0.17)	(0.61)
>50	− 0.00	− 0.44	− 0.43
	(0.23)	(0.35)	(0.81)
Female	0.06	0.23	0.35
	(0.13)	(0.14)	(0.46)
Unemployed	− 0.01	0.24*	5.10***
	(0.14)	(0.13)	(0.45)
*Residence region (ref: Eastern China)*			
Central China	− 0.19	0.06	1.00
	(0.20)	(0.18)	(0.62)
Western China	− 0.31*	0.07	0.42
	(0.16)	(0.16)	(0.53)
Northeast China	− 0.36**	0.16	0.97
	(0.18)	(0.20)	(0.73)
*Health status (ref: Good or better)*			
Fair	0.17	0.12	1.07*
	(0.19)	(0.17)	(0.57)
Poor	− 0.08	0.51**	0.87
	(0.25)	(0.21)	(0.76)
Constant	10.57***	7.81***	2.66***
	(0.19)	(0.20)	(0.57)
Observations	439	407	358
R^2^	0.02	0.06	0.41

∗∗∗, ∗∗, and ∗ denote the significance at the 1%, 5%, and 10% levels, respectively. Robust standard errors are reported in parentheses. Direct medical costs, direct non-medical costs, and indirect costs were log-transformed

Unemployment was significantly associated with increased direct non-medical costs (*P* <0.1) and indirect costs (*P* <0.01). Regional distinctions influenced direct medical costs, with Eastern China surpassing Northeast China (*P* <0.05), potentially reflecting better drug access. Direct non-medical and indirect costs, however, showed no regional variance.

In this study, we embarked on a comprehensive assessment of the disease burden and costs associated with MS in China. Our primary aim was to shed light on the intricate web of challenges faced by Chinese MS patients. To the best of our knowledge, this study is the first to use a patient self-report survey to comprehensively estimate the disease burden as well as the direct and indirect costs of MS in China. The results showed that the HRQoL of MS patients decreased dramatically as their disease progressed, and the mean utility of MS patients was 0.657. The average annual cost of MS for patients was 168,228 CNY (23,720 USD) per year, of which direct medical costs, direct non-medical costs, and indirect costs accounted for 36.8%, 23.6%, and 39.6%, respectively. Higher costs were found as the severity of disability increased, and the increase in the economic burden of MS was mainly driven by indirect costs.

The heavy burden of MS was reflected by the pattern of a decreasing HRQoL and an increasing unemployment rate with the disease progression in this study. Our findings are similar to those from previous research in European countries [12]. In terms of employment, previous studies in other countries have concluded that the onset of MS has a great impact on the productive years of patients and limits their employment and income [13]. The proportion of MS patients of working age who were unemployed or took early retirement due to MS was 47.6% in this study, higher than the 39% reported in the Global MS Employment Report 2016.

The medical costs of MS in China in published studies range from 5042.85 CNY (~711 USD) [10] to 27,655.57 CNY (~3899 USD) [9]. Our findings on direct medical costs suggest a higher economic burden than those revealed by other studies in China. The variation in costs is mainly due to the differences in the period and sources of collected data, the cost categories included, and the methodology of calculation. Specifically, the three previous studies using claims data or data collected by hospitals showed a similar low cost of MS, reflecting only the partial economic burden on MS patients, such as hospitalization costs and costs in claims data. Moreover, OoP expenditure in direct medical costs was high at the time of our study, although some disease-modifying therapy (DMT) drugs such as teriflunomide have been covered by the national health insurance scheme in China since 2020. The average annual OoP expenditure of 39,895 CNY (5625 USD) in this study was considerably higher than that reported for MS patients in Canada (1300 USD), or Norway (~1237 USD) [14]. With high OoP expenditure, the families of MS patients had a high risk of financial hardship, and thus additional policies targeting MS patients are required to alleviate their OoP costs.

Our findings suggested that the direct non-medical costs accounted for a considerable amount of the total cost (23.6%). In particular, the high economic burden of nutrition fees (defined as nutritional products other than the daily diet) due to MS may imply insufficient accessibility to a high-quality standard of care for MS patients in China. Studies have shown that dietary components and patterns potentially have a significant impact on MS [15]. This spotlight on the economic ramifications of nutrition fees, often overlooked in previous research, underscores potential challenges in delivering comprehensive care to Chinese MS patients.

Indirect cost was a significant component of the economic burden of MS patients, driving the costs associated with the disease, especially for patients with severe disabilities. Compared with studies performed in other countries, our findings affirmed the high proportion of indirect costs, which was similar to previous research using a societal perspective conducted in the United States [6]. The high indirect costs for MS patients stem from reduced productivity and employment disruptions during prime working years, alongside the substantial burden on informal caregivers. Disease-related impairments lead to absenteeism, lower efficiency, and unemployment, while caregivers often reduce their work commitments to provide support. This complex interaction underscores the pivotal role of employment dynamics and caregiving responsibilities in driving the elevated indirect costs linked to MS.

This study revealed an unbalanced utilization of professional care *versus* informal care (3.35% *vs.* 42.56%) for MS patients in China. The utilization of professional care by MS patients in China was lower than that in European countries. Previous studies have reported the utilization of formal care for MS patients to be 21.6% in the UK [12] and 36.2% in Spain [7]. Our results suggested an unmet need for professional care services by MS patients in China, and that the utilization of informal care was predominant to compensate for the lack of formal care. The unbalanced professional care utilization in China might be derived from factors such as inadequate nursing home facilities and insufficient coverage by relevant insurance policies for patients with rare diseases.

Our study also revealed the heavy economic burden of MS incurred by patients in terms of the distribution of disability severity. The total cost of MS increased with increased disability status, leading to a tremendous economic burden at the end-stage of the disease. Recent studies have focused on the relationship between total costs and disability severity, most of which concluded a positive association [5, 6]. In this study, we investigated direct medical costs, direct non-medical costs, and indirect costs as separate components, and found a significant association between indirect costs and disability severity, while the insignificant association of direct costs with disability severity may result from the decreased DMT utilization.

MS presents unique challenges that extend beyond the usual parameters of healthcare expenditure. As a rare disease in China, there are added difficulties in both diagnosis and treatment, often requiring more resources to reach effective treatment compared to common diseases. Moreover, MS affects the nervous system, leading to long-term disability, and thus imposes a greater burden on long-term care and productivity loss.

Our study has several limitations. First, relying on cross-sectional self-reported data may introduce biases like perception bias and recall errors. Second, while the participant demographics in this study aligned with the MS epidemiology in China, our findings might not be fully representative of the broader MS patient population in China due to the non-randomized recruitment process. Regional imbalance in online recruitment due to the COVID-19 pandemic further limits the generalizability of our results. In addition, the absence of educational data from our study participants could influence the interpretation of HRQoL and cost components. Therefore, future research should aim to utilize nationally representative on-site surveys or integrate multiple data sources to provide a more comprehensive validation of the economic burden of MS in China.

In sum, our study demonstrated that MS induces both a heavy disease burden and high costs for patients and their families. In addition, we found that more severely impaired HRQoL and higher costs were imposed on MS patients as the disability severity increased. Despite the increasing attention on policy regarding rare diseases and the greater availability of DMT drugs for MS in China, the heavy burden and costs for MS patients revealed in our study should be considered for the design of future interventions. It is essential that policymakers adopt a holistic view of the economic burden of MS, including factors such as productivity losses for patients and their caregivers.

### Supplementary Information

Below is the link to the electronic supplementary material.

Supplementary file1 (PDF 235 KB)

## Data Availability

The original contributions presented in the study are included in the article material, further inquiries can be directed to the corresponding authors.
