# Supplementary material for: Disease Burden and Costs Associated with Multiple Sclerosis in China: A Cross-sectional Analysis of Nationwide Survey Data
Source: Neurosci Bull. 2023 Nov 2;40(4):533–8. doi: 10.1007/s12264-023-01135-5 (PMC11003946; doi:10.1007/s12264-023-01135-5)
Supplement: Supplementary file 1 — Supplementary file1 (PDF 235 KB) [file 12264_2023_1135_MOESM1_ESM.pdf]

Supplementary Material

Methodology Notes

Study Design

This was a cross-sectional study conducted in June 2020 in China. Due to the COVID-19 pandemic, we used a web-based self-report questionnaire to collect information from MS patients concerning demographics, resource utilization, and costs. All study participants were invited and recruited through an online MS community that was informally organized and consisted of >2,000 MS patients. The inclusion criteria were that patients were clinically diagnosed with MS and consented to participate. All participants were required to upload electronic copies of confirmed MS diagnosis records after completing the survey for quality check. Patients' family members were allowed to complete the questionnaire on behalf of the patients except for the questions regarding health utility.

To ensure data quality and questionnaire authenticity, we applied rigorous protocols, including real-time monitoring of completion times for each survey, as well as restricting participation exclusively to registered members of a recognized MS patient community. In addition, to confirm the accuracy of MS diagnoses among our participants, we cross-verified patients' diagnostic records and associated MS treatment prescriptions as a quality control measure.

Study Data and Measures

The questionnaire had 21 questions aiming to obtain information on MS patients, including demographic data (e.g., age, gender, city of residence, and employment status), health status and HRQoL, resource utilization (e.g., use of disability aids, and professional and informal care), and costs (direct medical costs, direct non-medical costs, indirect costs, and out-of-pocket [OoP] payments).

Self-reported health status was evaluated using a 5-level scale consisting of “excellent”, “great”, “good”, “fair”, and “poor”. The EQ-5D-5L was used to describe HRQoL according to 5 levels (no problems or slight, moderate, severe, and extreme problems) [1]. The health utility was calculated based on the EQ-5D-5L value set for China [2].

The expanded disability status scale (EDSS) is widely used to assess the degree of neurological impairment in MS [3]. However, it requires an assessment by a professional practitioner. In this study, disease progression status was measured indirectly using a question to describe a patient’s current status, with options consisting of 5 levels as a proxy for the EDSS score (EDSS <4: “able to walk with no restriction or disorder”; EDSS 4–6: “able to walk but with functional system disorder such as vision, sensory or motor” *or* “only able to walk with crutches”; EDSS >6: “activities limited to wheelchairs” *or* “sick in bed”). The measure of the self-reported level of disability is aligned with a previous study on the Self-Reported Disability Status Scale [4].

Direct costs consisted of direct medical costs and direct non-medical costs. Direct medical costs included diagnosis, treatment, and drug costs incurred in both outpatient and inpatient settings, and were clearly defined and explicitly collected from respondents *via* the question concerning “the total medical cost in the past year”. Direct non-medical costs included adaptation costs (e.g., crutches and wheelchairs), costs of professional care received (non-medicals services for daily living aids), related nutrition fees, and transportation costs to healthcare facilities. The former two were calculated by asking patients to provide information on “buying a crutch or wheelchair” and “hiring a professional caregiver for daily living aids” together with the corresponding costs. The costs of nutrition and transportation were clearly defined and collected by questions concerning costs incurred. In addition, we also asked respondents to provide information on “out-of-pocket payments in the past year” to calculate the OoP expenditure as a share of the total direct medical costs.

Indirect costs refer to the time lost and related productivity losses for both MS patients and informal caregivers. Informal caregivers are defined here as family members or relatives. MS patients' indirect costs were calculated based on the reduction of the monthly or annual income of patients and their family members. The average income for calculating productivity loss refers to the GDP per capita in 2020 in China, which was 72,000 yuan per year (197.26 yuan per day) [5]. All these costs were included in the calculation of average annual costs for MS patients. Costs were calculated in Chinese yuan (CNY) and US dollars (USD), and were based on the exchange rates of the State Administration of Foreign Exchange of China, expressed in US dollars (1 CNY equivalent to 0.141 USD on June 30, 2020) [6].

Statistical Analysis

Descriptive statistics (frequency, mean, and standard deviation [SD]) are reported for resource utilization and costs. The χ^2 test was used to examine the differences in demographic factors across levels of disease severity. We used ordinary least square (OLS) estimation for the log transformation of annual direct medical costs, direct non-medical costs, and indirect costs, since cost data were often not normally distributed and were usually transformed for regression analysis [7]. The estimation model between costs and disease severity can be written as follows:

$$Y_i = \alpha_0 + \delta EDSS_i + X_i\beta + \varepsilon_i$$

The subscript i indicates an individual. Y_i represents the dependent variables (log-transformed direct medical cost and indirect cost). X_i is a vector of demographic and contextual variables, including age, gender, employment status, residence region, and self-reported health status. The key variable of interest is $EDSS_i$, a vector of dummy variables representing the disability severity that is derived from the simplified 3-category variable as a proxy for the EDSS score. The coefficient δ captures the association between costs and the level of disease severity. Stata/SE 17 was used for statistical analyses.

Descriptive Statistics Notes

Characteristics of Patients with MS

MS patients in the study were predominantly female (65.6%), with a mean age of 34.5 years, which is aligned with previous epidemiological research showing a female-to-male ratio of 2.02 in China [8]. Most patients (66.7%) had a status of mild disability (EDSS <4).

Table 1 Characteristics of study participants

**Variables**	**Sample (*n* = 477)**	**EDSS <4 (*n* = 318)**	**EDSS 4–6 (*n* = 130)**	**EDSS >6 (*n* = 29)**	χ^2	***P*-value**
Proportion of responses answered by family members as a proxy, n (%)	70 (14.68)	42 (13.21)	21 (16.15)	7 (24.14)	2.848	0.241
**Age, years**						
Mean (SD)	34.5 (10.26)	32.6 (8.79)	37.6 (12.04)	40.8 (10.98)		
<30, n (%)	198 (41.51)	151 (47.48)	43 (33.08)	4 (13.79)	35.293	<0.001
31–40, n (%)	163 (34.17)	111 (34.91)	41 (31.54)	11 (37.93)		
41–50, n (%)	85 (17.82)	46 (14.47)	29 (22.31)	10 (34.48)		
>50, n (%)	31 (6.50)	10 (3.14)	17 (13.08)	4 (13.79)		
**Gender**						
Male, n (%)	164 (34.38)	110 (34.59)	42 (32.31)	12 (41.38)	0.884	0.643
Female, n (%)	313 (65.62)	208 (65.41)	88 (67.69)	17 (58.62)		
**Residence region**						

Eastern China, n (%)	211 (44.23)	152 (47.8)	46 (35.38)	13 (44.83)	8.413	0.209
Central China, n (%)	77 (16.14)	49 (15.41)	23 (17.69)	5 (17.24)		
Western China, n (%)	127 (26.62)	81 (25.47)	41 (31.54)	5 (17.24)		
Northeast China, n (%)	62 (13.00)	36 (11.32)	20 (15.38)	6 (20.69)		
**Working status**						
Employed, n (%)	240 (50.31)	185 (58.18)	52 (40.00)	3 (10.34)	54.029	<0.001
Unemployed, n (%)	187 (39.20)	101 (31.76)	65 (50.00)	21 (72.41)		
Retired, n (%)	18 (3.77)	6 (1.89)	7 (5.38)	5 (17.24)		
Student, n (%)	32 (6.71)	26 (8.18)	6 (4.62)	-		
**Productivity loss of MS patients**						
Proportion of MS patients of working age, n (%)	460 (96.44)	316 (99.37)	119 (91.54)	25 (86.21)	25.872	<0.001
Unemployment or early retirement due to MS, n (%)	228 (47.80)	119 (37.42)	83 (63.85)	26 (89.66)	47.504	<0.001
Absence days due to MS if employed, mean (SD)	63.5 (45.41)	62.7 (43.42)	62.4 (49.99)	-	-	-
**Self-reported health status**						
Good, great, or excellent, n (%)	138 (29.11)	125 (39.68)	13 (10.00)	-	152.787	<0.001
Fair, n (%)	234 (49.37)	169 (53.65)	59 (45.38)	6 (20.69)		
Poor, n (%)	102 (21.52)	21 (6.67)	58 (44.62)	23 (79.31)		
**HRQoL and utility**						
EQ-5D VAS, mean (SD)	61.0 (23.25)	68.0 (20.36)	48.6 (21.39)	35.1 (22.20)	0.673	0.714

Health utility, mean (SD)	0.657 (0.302)	0.808 (0.137)	0.437 (0.277)	0.017 (0.256)	106.850	<0.001

Note: Patients were considered employed if they were hired full-time or part-time. Health utility value was calculated based on the EQ-5D-5L questionnaire using the EQ-5D-5L value set for China. EDSS, expanded disability status scale; SD, standard deviation; HRQoL, health-related quality of life; VAS, visual analog scale.

Resource Utilization Due to MS

The proportion of MS patients using disease-modifying therapies (DMTs) was 49.1%, and as the disease progressed, the usage of DMTs decreased. Concerning the use of disability aids, the proportions of patients requiring the use of wheelchairs and crutches were 17.9% and 20.0% (Table 2). The proportion of wheelchair use among patients with EDSS <4 was 5.1%, whereas for patients with EDSS >6 it was 96.6%, similar to that of crutch use. The utilization of professional care was very low (3.4%), and an average of 5.5 months of professional care was needed per year.

Regarding the utilization of informal care, 42.6% of MS patients received home care services from family members or relatives at the time of the study. With increasing disability, a greater proportion of patients required informal care (EDSS <4: 28.3%; EDSS 4–6: 67.7%; EDSS >6: 86.2%). The average days of informal care per month was 19.5. The data showed a similar trend, that patients required more days per month of informal care with increasing disability (EDSS <4: 16.4 days; EDSS 4–6: 19.7 days; EDSS >6: 28.9 days).

Table 2 Resource utilization due to MS

Resource utilization and productivity loss	Sample (*n* = 477)	EDSS<4 (*n* = 318)	EDSS 4-6 (*n* = 130)	EDSS>6 (*n* = 29)
**DMT drug utilization, %**	49.1	53.1	43.8	27.6
**Disability aids**				
Proportion of patients using wheelchairs, %	17.9	5.0	31.8	96.6
Proportion of patients using crutches, %	20.0	6.0	40.3	82.8
**Professional care**				
Proportion of patients receiving services, %	3.4	2.2	6.2	3.5
Average month of professional care needed among patients receiving professional care, n	5.5	1.3	8.2	2.0
**Informal care**				
Receiving home care services, %	42.6	28.3	67.7	86.2
Days of informal care utilization per month, n	19.5	16.4	19.7	28.8

Notes: DMT refers to disease-modifying therapies. DMT drugs in the survey included fingolimod, teriflunomide, and human interferon beta-1b, which were three DMT drugs listed in China at the time of the survey conducted in June 2020.

References

[1] Herdman M, Gudex C, Lloyd A, *et al*. Development and preliminary testing of the new five-level version of EQ-5D (EQ-5D-5L). Quality of Life Research, 2011, 20(10): 1727–1736.

[2] Luo N, Liu G, Li M, *et al*. Estimating an EQ-5D-5L Value Set for China[J]. Value in Health: The Journal of the International Society for Pharmacoeconomics and Outcomes Research, 2017, 20(4): 662–669.

[3] Kurtzke J F. Rating neurologic impairment in multiple sclerosis: An expanded disability status scale (EDSS). Neurology, Wolters Kluwer Health, Inc. on behalf of the American Academy of Neurology, 1983, 33(11): 1444–1444.

[4] Kaufmann M, Salmen A, Barin L, *et al*. Development and validation of the self-reported disability status scale (SRDSS) to estimate EDSS-categories[J]. Multiple Sclerosis and Related Disorders, 2020, 42: 102148.

[5] National Bureau of Statistics of China. China Statistical Yearbook 2021[EB/OL]. 2021/2021-09-30. http://www.stats.gov.cn/tjsj/ndsj/2019/indexeh.htm.

[6] State Administration of Foreign Exchange of China. Conversion rate table of various currencies to U.S. dollar[EB/OL]. 2020/2021-11-23. http://www.safe.gov.cn/safe/2020/0630/16541.html.

[7] Thompson S G, Barber J A. How should cost data in pragmatic randomised trials be analysed? BMJ, British Medical Journal Publishing Group, 2000, 320(7243): 1197–1200.

[8] Tian D-C, Zhang C, Yuan M, *et al*. Incidence of multiple sclerosis in China: A nationwide hospital-based study. The Lancet Regional Health. Western Pacific, 2020, 1: 100010.

Multiple Sclerosis Burden of Disease Questionnaire[1]

Dear Patient:

Hello! We are researchers at the School of Public Health at Fudan University, and you are invited to participate in this project: Burden and Costs of Multiple Sclerosis.

In order to understand the disease burden of patients with multiple sclerosis, and improve the medical security of patients with multiple sclerosis and the support of the medical service system for patients, together with the School of Public Health of Fudan University and the Cord Center for Rare Diseases (CORD, the former Center for Rare Disease Development), Multiple Sclerosis Home is cooperating with this survey to understand the patient's physical condition, and the financial burden of multiple sclerosis treatment on patients or their caregivers.

The subjects of this survey are patients with multiple sclerosis. The content of the questionnaire includes three parts: the basic situation of the patient, the cost of the patient's multiple sclerosis treatment, and the patient's health status. We are asking for the patient, or the patient's family, to please complete the survey. This survey will take you about 10 minutes. Participation in the survey is completely voluntary. Please fill in the questionnaire completely according to your actual situation. The data and information obtained will be completely confidential and will only be used for scientific research.

You can voluntarily choose to participate or not to participate in this research project, and you can withdraw from this research at any time without any consequences. Participation in this research also does not prejudice any rights that you have. If you have any questions about this research, you can contact the research group (Dr. Hu, mobile phone number: xxxxxxxxx). Thank you very much for your support!

Would you like to take part in the survey? [multiple choice] *

○ Yes

○ I am not willing to (please skip to the end and submit the answer sheet)

1. Is the survey being answered by the patient herself/himself [multiple choice] *

○ Answered by the patient

○ Answered on behalf of the patient by a family member (Please provide the information below according to the patient)

2. Your age is: _____ years [please fill in the blank]

3. Your gender is: [multiple choice] *

[1] English language translation of original questionnaire in Mandarin Chinese.

○ Male ○Female

4.Your permanent residence is: [please fill in the blank] *

_______Province __________City

5. Your current work status is: [multiple choice] *

○ Employed (including full-time and part-time)

○ Retired

○ Unemployed

○ Student

6. Your current physical condition in most cases is: [multiple choice] *

○ Unrestricted walking, mild impairment of functional systems such as vision, sensory or motor, but not affecting daily activities

○ Walking is not limited, but there are serious impairments in functional systems such as vision, sensory or motor systems, which will affect daily activities

○ Need to use crutches to walk

○ Activities are limited to wheelchair, using crutches to walk no more than 5 meters

○ Sick in bed

○ I don't know

7. Do your current multiple sclerosis drugs include the following DMT drugs? [multiple choice] *

□ Fingolimod (Gilenya®)

□ Teriflunomide (Aubagio®)

□ Recombinant human interferon beta-1b for injection (Betaferon®)

□ None

□ Don't know/Not clear

8. Have you purchased crutches because of multiple sclerosis? [multiple choice] *

○ No

○ Yes

9. Have you purchased a wheelchair because of multiple sclerosis? [multiple choice] *

○ No

○ Yes

10. In an average month, your nutritional expenses (nutritional products other than your daily diet) due to multiple sclerosis are about ____________ yuan/month [please fill in the blank]

11. In an average month, your transportation expenses for multiple sclerosis treatment are about ____________ yuan/month [please fill in the blank]

12. In an average year, your additional accommodation expenses for multiple sclerosis treatment are about ______ yuan/year [please fill in the blank]

13. Have you been on sick leave, unemployed, or retired early for more than a year because of multiple sclerosis? [multiple choice]

○ No

○ Yes (please skip to question 14)

13-1. Have you been on sick leave, unemployed, or retired for less than one year due to multiple sclerosis? [multiple choice]

○ No

○ Yes, the average number of days lost in a year is: _________________ *

14. Do you utilize nursing care for multiple sclerosis? [multiple choice]

○ No (please skip to question 15)

○ I hire a professional caregiver (please skip to question 14-1)

○ I have my family member/relatives caring for me (please skip to question 14-2)

○ I have both professional and family caregivers (please skip to question 14-3)

14-1. The average nursing time of professional care is ______ months/year, and the monthly salary of the professional caregiver is ________ yuan/month [please fill in the blank]

14-2. In an average month, the number of days of family care is about ______ days/month [please fill in the blank]

14-3. The average nursing time of professional care in one year is ______ months/year, the monthly salary of the professional caregiver is ________ yuan/ month, and the number of days of family care in an average month is about ______ days/month [please fill in the blanks]

15. The total cost of outpatient, hospitalization, medicine, etc. for the treatment of multiple sclerosis in an average year is about ____________ yuan/year (including the expenses paid by various medical insurances). Among them, for the individual, after deducting medical insurance assistance, the out-of-pocket payment is ______ yuan/year [please fill in the blank]

Please select the statement below that best describes your current physical condition. (Please ask the patient to answer if you are a family member)

16. Mobility (walking) today: [multiple choice]

○ I have no problems in walking about

○ I have slight problems in walking about

○ I have moderate problems in walking about

○ I have severe problems in walking about

○ I am unable to walk about

17. Self-care (dressing and washing) today: [multiple Choice]

○ I have no problems washing or dressing myself

○ I have slight problems washing or dressing myself

○ I have moderate problems washing or dressing myself

○ I have severe problems washing or dressing myself

○ I am unable to wash or dress myself

18. Usual activities (working, reading, or housework) today: [multiple choice]

○ I have no problems doing my usual activities

○ I have slight problems doing my usual activities

○ I have moderate problems doing my usual activities

○ I have severe problems doing my usual activities

○ I am unable to do my usual activities

19. Pain or discomfort: [multiple choice]

○ I have no pain or discomfort

○ I have slight pain or discomfort

○ I have moderate pain or discomfort

○ I have severe pain or discomfort

○ I have extreme pain or discomfort

20. Anxiety or depression: [multiple choice]

○ I am not anxious or depressed

○ I am slightly anxious or depressed

○ I am moderately anxious or depressed

○ I am severely anxious or depressed

○ I am extremely anxious or depressed

21. In general, your health status is: [multiple choice]

○ Excellent

○ Great

○ Good

○ Fair

○ Bad

22. We would like to know how good or bad your health is TODAY. This slider represents from 0 to 100. 100 represents the best health you can imagine, 0 represents the worst health you imagine, please indicate on the slider how your health is today.

[Enter a number from 0 (the worst health you can imagine) to 100 (the best health you can imagine).]

____

23. Please kindly take a photo to upload of your multiple sclerosis medical record report or the diagnosis records [upload file title]
